# Geniposide Alleviates Amyloid-Induced Synaptic Injury by Protecting Axonal Mitochondrial Trafficking

**DOI:** 10.3389/fncel.2016.00309

**Published:** 2017-01-25

**Authors:** Haijing Zhang, Chunhui Zhao, Cui Lv, Xiaoli Liu, Shijing Du, Zhi Li, Yongyan Wang, Wensheng Zhang

**Affiliations:** ^1^Beijing Area Major Laboratory of Protection and Utilization of Traditional Chinese Medicine, Beijing Normal UniversityBeijing, China; ^2^College of Life Science, Beijing Normal UniversityBeijing, China; ^3^Engineering Research Center of Natural Medicine, Ministry of Education, Beijing Normal UniversityBeijing, China; ^4^Institute of Chinese Materia Medica, China Academy of Chinese Medical SciencesBeijing, China; ^5^Laboratory of Immunology for Environment and Health, Shandong Analysis and Test Center, Shandong Academy of ScienceJinan, China; ^6^College of Resources Science Technology, Beijing Normal UniversityBeijing, China; ^7^Engineering Research Center of Sanqi Biotechnology and PharmaceuticalKunming, China

**Keywords:** axonal transport, mitochondrial trafficking, amyloid β, geniposide, synaptic loss, spine morphology

## Abstract

Synaptic and mitochondrial pathologies are early events in the progression of Alzheimer's disease (AD). Normal axonal mitochondrial function and transport play crucial roles in maintaining synaptic function by producing high levels of adenosine triphosphate and buffering calcium. However, there can be abnormal axonal mitochondrial trafficking, distribution, and fragmentation, which are strongly correlated with amyloid-β (Aβ)-induced synaptic loss and dysfunction. The present study examined the neuroprotective effect of geniposide, a compound extracted from gardenia fruit in Aβ-treated neurons and an AD mouse model. Geniposide alleviated Aβ-induced axonal mitochondrial abnormalities by increasing axonal mitochondrial density and length and improving mitochondrial motility and trafficking in cultured hippocampal neurons, consequently ameliorating synaptic damage by reversing synaptic loss, addressing spine density and morphology abnormalities, and ameliorating the decreases in synapse-related proteins in neurons and APPswe/PS1dE9 mice. These findings provide new insights into the effects of geniposide administration on neuronal and synaptic functions under conditions of Aβ enrichment.

## Introduction

Mitochondrial damage and synaptic dysfunction are early events in the pathogenesis of Alzheimer's disease (AD; Reddy and Beal, 2008; Hauptmann et al., 2009; Reddy, 2009; Reddy et al., 2012). Synapses, which are the basic structural foundations of signal transduction in the central nervous system, form connections, and transmit chemical signals among neurons (Billups and Forsythe, 2002; Li et al., 2004). Severe structural and functional damage to synapses fundamentally cause cognitive and memory dysfunctions (Du et al., 2008; Adalbert and Coleman, 2013). Changes in the density and morphology of synapses and dendritic spines can be detected in the cerebral cortex and hippocampus of AD patients (DeKosky et al., 1996). Cognitive dysfunction is more strongly correlated with synaptic loss than with senile plaques, neurofibrillary tangles, neural loss, or gliosis (Pozueta et al., 2013).

Synapses require a large amount of energy provided by mitochondria, which involves mitochondrial fusion, fission, and transport from the soma. Mitochondria are distributed throughout neurons, including at synapses, via transfer through axons and dendrites. The proper intracellular distribution of the mitochondria is critical for the normal physiological functions of neuronal cells (Cai and Tammineni, 2016), such as neurotransmission, synaptic plasticity, and axonal outgrowth (Li et al., 2004). Abnormal distribution and transport disorders of the mitochondria can be observed in AD models (Stokin et al., 2005; Du et al., 2010; Sheng and Cai, 2012; Umeda et al., 2015) and influence the synthesis of neurotransmitters, the release of synaptic vesicles, and calcium homeostasis, ultimately resulting in synaptic dysfunction and adenosine triphosphate (ATP) deficiency (Billups and Forsythe, 2002; Hollenbeck, 2005; Verstreken et al., 2005). In this process, amyloid-β (Aβ) is regarded as the major toxic molecule (Rui et al., 2006; Du et al., 2010).

Aβ is transported to the mitochondria via receptor for advanced glycation end products (RAGE; Takuma et al., 2009), the translocase of the outer membrane (TOM) machinery (Hansson Petersen et al., 2008; Reddy, 2009), or endoplasmic reticulum–mitochondrial crosstalk (Hedskog et al., 2013). Aβ progressively accumulates in the neuronal mitochondria of AD mouse models overexpressing Aβ, AD brains, and cultured neurons (Manczak et al., 2006, 2011; Reddy and Beal, 2008; Reddy, 2009; Calkins et al., 2011). The accumulation of Aβ in the mitochondria occurs before the deposition of extracellular Aβ plaques and increases with age, particularly in synaptic mitochondria, which are vulnerable to cumulative damage, and induces the exaggeration of synaptic and mitochondrial injuries, such as the overproduction of reactive oxygen species (ROS; Manczak et al., 2006), decreased ATP, and hypometabolism (Cardoso et al., 2004; Du and Yan, 2010). Recent studies have shown that neuronal axonal mitochondria exhibited significantly repressed mobility and increased fragmentation (Du et al., 2010; Calkins and Reddy, 2011). Increasing evidence suggests that Aβ-induced synaptic mitochondrial dysfunction contributes to synaptic injury (Du et al., 2008, 2010; Calkins et al., 2011; Calkins and Reddy, 2011; Fang et al., 2015). Thus, strategies to protect synaptic mitochondria against the structural and functional damage of Aβ may effectively prevent the deterioration of synaptic mitochondrial dysfunction and halt AD progression.

Geniposide (Zhao et al., 2016a), an iridoid glucoside compound isolated from gardenia fruit (*Gardenia jasminoides Ellis*, Rubiaceae), attenuated the oligomeric Aβ_1–42_-induced mitochondrial dysfunction by restoring ATP generation, mitochondrial membrane potential, and cytochrome c oxidase and caspase-3/-9 activity by reducing ROS production and cytochrome c leakage, as well as by inhibiting apoptosis (Lv et al., 2014a, 2015). Most studies also focused on the neuroprotective effect of geniposide against brain diseases, especially neurodegenerative disorders (Gao et al., 2014; Liu et al., 2015; Zhang et al., 2015). However, the protective effect of geniposide on axonal mitochondrial trafficking and synaptic injury in neurons remains unclear. The outcome of this study on cultured neurons showed that geniposide treatment significantly alleviates Aβ-induced synaptic structural and morphological injuries by protecting axonal mitochondrial trafficking and morphology.

## Materials and methods

### Chemicals

Geniposide (Zhao et al., 2016b; purity > 98%) was purchased from the National Institute for the Control of Pharmaceutical and Biological Products (Beijing, China) and was free of endotoxins. 1,1,1,3,3,3-Hexafluoro-2-propanal (HFIP) and penicillin/streptomycin were obtained from Sigma (St. Louis, MO, USA). Fetal bovine serum (FBS), B27, and Neurobasal-A medium were purchased from Gibco (Waltham, MA, USA).

### Mice and drug administration

Experimental mPrP-APPswe/PS1dE9 (APP/PS1) doubly transgenic mice and C57BL/6 mice were purchased from Beijing HFK Bio-Technology Co., Ltd. Nine-month-old male mice were individually housed under a 12 h light/dark cycle at an ambient temperature of 23 ± 1°C and relative humidity of 55 ± 5% and were given food and water *ad libitum*. Before the experiment, the mice were housed under these conditions for 2–3 days to allow them to adapt to the environment. APP/PS1 mice were randomly categorized into the treatment groups or the vehicle group and treated with either geniposide (12.5, 25, or 50 mg/kg/days; *n* = 15) or water (*n* = 15), respectively, for 3 months via intragastric administration. Age-matched C57BL/6 mice were fed water as the vehicle control (*n* = 15). Geniposide was dissolved in water within 24 h before use. Equal volumes of liquid were given to each group daily for 3 months before the mice were sacrificed.

All animal procedures performed in this study were approved by the Beijing Normal University Laboratory Animal Care and Use Committee in accordance with the National Institute of Health “Guidelines for the Care and Use of Laboratory Animals” (NIH Publications No. 8023, revised 1996). The mice were sacrificed by cervical dislocation after being anesthetized. All efforts were made to minimize the number of animals used and their suffering.

### Oligomeric Aβ_1–42_ preparation

Oligomeric Aβ_1–42_ was prepared from commercially available synthetic peptides (Sigma Chemical Co., St. Louis, MO, USA), as previously described (Dahlgren et al., 2002; Yin et al., 2011). The lyophilized peptide was resuspended in cold HFIP at a concentration of 1 mg/mL and aliquoted into microcentrifuge tubes to quickly obtain 0.1 mg stocks. The stocks were stored at room temperature and protected from light for 2–4 h before the removal of HFIP under gentle vacuum, thereby leaving a thin transparent film of peptides on the internal surface of the tube. The stocks were stored at −20°C. For the aggregation protocols, HFIP-treated peptides were dissolved in anhydrous dimethyl sulfoxide at 5 mM and diluted to 100 μM in Ham's F12 Nutrient Mixture (Thermo Fisher Scientific, Waltham, MA, USA). The diluted peptides were incubated at 4°C for 24 h to obtain oligomeric Aβ_1–42_.

### Hippocampal neuronal culture and treatment

One-day-old male C57BL/6 mice were purchased from Beijing Vital River Laboratory Animal Technology Co., Ltd., and transported within a specific pathogen-free, air permeable, and bacteria shield shipping box. The mice were sacrificed by cervical dislocation.

Primary hippocampal neurons were prepared from the hippocampi of 1 day-old (newborn) pups. The hippocampi were dissected in cold D-Hanks solution. Tissues were collected and washed in D-Hanks, and 0.05% (v/v) trypsin was added for digestion at 37°C for 20 min. Digestion was terminated by adding FBS to a final concentration of 10% (v/v). Cells were collected by centrifugation at 800 × g for 10 min to remove the D-Hanks solution and were then resuspended in Neurobasal-A medium (Thermo Fisher Scientific, Waltham, MA, USA) supplemented with 2% (v/v) B27 (Thermo Fisher Scientific).

For the various analyses, cells were plated onto 6-, 12-, and 96-well-plates or glass-bottom dishes with four chambers (CELLview, Greiner, Germany) (~5 × 10^4^ cells/mL) pre-coated with poly-D-lysine (10 μg/mL). The cells were cultured at 37°C and 5% CO_2_ until use. The initial medium was removed after 4 h and replaced with fresh medium. After 14 days, the medium was replaced with Neurobasal-A medium without serum and phenol red (which affect the aggregation of Aβ). The primary cultured hippocampal neurons were pre-incubated for 24 h in the absence or presence of geniposide (2.5, 5, or 10 μM) before adding oligomeric Aβ_1–42_ (200 nM) for 24 h to assess the protective effect of geniposide on the Aβ_1–42_-treated neurons.

### Western blot assay

The brain tissue or neuron samples were lysed in 10 volumes (w/v) of radio-immunoprecipitation assay buffer containing a cocktail of complete protease and phosphatase inhibitors and were centrifuged at 15,000 × g for 10 min at 4°C. The protein concentration of the supernatant was determined using the BCA method. Proteins were examined by Western blot analysis using standard protocols. Equal amounts of proteins were loaded and resolved by 10% sodium dodecyl sulfate polyacrylamide gel electrophoresis and transferred to nitrocellulose membranes (Millipore, Billerica, MA, USA). The membranes were incubated in blocking solution (5% skim milk in PBST, 20 mM Tris-HCl, 150 mM NaCl, 0.1% Tween-20) at room temperature for 1.5 h. The membranes were incubated and gently shaken overnight (at 4°C) in PBST containing 5% skim milk and the indicated primary antibodies from among the following: monoclonal rabbit antibodies against c-AMP response element binding protein (CREB, 1:400, Cell Signaling, Beverley, MA, USA), and GAPDH (1:3000, Cell Signaling); polyclonal rabbit antibodies against phosphorylated Ca^2+^/calmodulin dependent protein kinase II α (p-CaMKIIα, 1:8000, Santa Cruz, CA, USA); and monoclonal mouse antibodies against synaptophysin (1:500, Abcam, Cambridge, UK), CaMKIIα (1:250, Santa Cruz), phosphorylated CREB (p-CREB, 1:400, Cell Signaling), postsynaptic density protein-95 (PSD-95, 1:500, Abcam), and β-actin (1:2000, Santa Cruz). After four washes with PBST, the membranes were incubated for 1.5 h at room temperature with the corresponding secondary antibodies. The membranes were washed four times with PBST and detected with an infrared imaging system (Odyssey). The intensity of the blots was analyzed and compared using NIH ImageJ program.

### ROS measurement

ROS were measured with 2′,7′-dichlorodihydrofluorescein diacetate (DCFH-DA). The cell-permeable DCFH-DA can be oxidized to dichlorofluorescein (DCF) by ROS in the cytoplasm and emit intensely fluorescence. After incubation with Aβ_1–42_ in the presence and absence of geniposide for 24 h, cultured neurons were washed with PBS and incubated with 10 μM DCFH-DA for 30 min at 37°C in an incubator with 5% CO_2_. Fluorescence images were captured using a laser-scanning confocal microscope (TCS-SPE, Leica, Germany). Fluorescence intensity was analyzed using NIH ImageJ program.

### Axonal mitochondrial density assay and length measurement

Hippocampal neurons at 14 day *in vitro* (DIV) were used for axonal mitochondrial density assay, and the length of these neurons was measured after the treatments. Neurons cultured in glass-bottom dishes with four chambers (CELLview, Greiner, Germany) were incubated with MitoTracker Red (Thermo Fisher Scientific, Waltham, MA, USA) at 100 nM for 20 min at 37°C and 5% CO_2_. Neurons were fixed in 4% paraformaldehyde for 20 min after being permeabilized with 0.5% Triton X-100 in 0.1% sodium citrate and blocked with 10% goat serum. Up to 0.25 mL of anti-Tau antibody (Abcam, 1:500) was added to each chamber, followed by incubation with goat anti-rabbit secondary antibody (Alexa Fluor 488, Abcam, UK). Axons were identified based on morphological characteristics. In live images, branches that are longer and with thin and uniform diameter, as well as sparse branching, were considered to be axons. Particles with strong red fluorescence (compared with background) and clear edges colocalized with axons were considered to be mitochondria. Fluorescence images were captured using a laser-scanning confocal microscope (TCS-SPE, Leica, Germany) and analyzed using NIH ImageJ program.

### Recording and analysis of axonal mitochondrial trafficking

MitoTracker Green (Thermo Fisher Scientific, Waltham, MA, USA) was used to label the mitochondria in living neurons. The axons and mitochondria were identified using the procedure described in Section Axonal Mitochondrial Density Assay and Length Measurement. The proximal region of axons was selected for time-lapse imaging analysis. Time-lapse images were captured under an inverted laser-scanning confocal microscope (TCS-SPE, Leica, Germany) with a stage-based chamber under 5% CO_2_ and 37°C. Time-lapse image stacks composed of five images (512 × 512 pixel) were taken every 10 s for 5 min for a total of 150 images under 40× magnification. Kymographs were generated using the kymograph tool for ImageJ program under maximum intensity projection. The width of the kymographs represents the length (μm) of the imaged axon, and the height represents the recording time. A mitochondrion was considered to be stationary if it remained non-mobile for the entire recording period. A mitochondrion was considered movable only if the displacement was more than 2 μm. The percentages of stationary and movable mitochondria and of anterograde and retrograde mitochondrial movement were measured separately from the corresponding kymographs by using NIH ImageJ program. A mitochondrion that moves from the soma toward the distal end of an axon is considered anterograde, whereas movement from the distal end toward the soma is considered retrograde. The average velocity (μm/s) of all anterograde and retrograde axonal mitochondria was calculated using the displacement of each mitochondrion.

### Dendritic spine density measurement and morphological assay

Neurons cultured in glass-bottom dishes with four chambers (CELLview, Greiner, Germany) were fixed in 4% paraformaldehyde for 20 min and washed with PBS after the neurons were incubated with 2 μM preheated CellTracker CM-DiI (Life Technologies, Waltham, MA, USA) for 20 min at 37°C. LAS X software was used to control the laser-scanning confocal microscope (TCS-SPE, Leica, Germany) equipped with a 63× objective and excitation at 543 nm. Images were taken under the confocal microscope, and Z-stacks were gathered at increments of 0.67 μm. To increase the accuracy of the identification of the surfaces of the dendrite shaft and spines, sequence images were deconvolved to reduce point-spread functions by using the adaptive blind 3D deconvolution method (AutoQuant X, Media Cybermedics, Inc., Bethesda, MD) prior to analysis. The output images were obtained with Build Neurites and Build Spines in NeuronStudio software (CNIC, Mt. Sinai School of Medicine, New York, USA) to mark and record all the spines on the selected dendrites. Dendritic protrusions with a clearly identifiable neck attached to the branch of the dendrite composed a spine. The output data included length, neck diameter, and head diameter of each spine, based on which the types of spines were recognized. Spines with a spine length-to-neck diameter ratio <2.0 were defined as of the stubby type. Spines were categorized as mushroom type if they presented a length-to-neck diameter ratio of more than 2.0 and a head-to-neck diameter ratio of more than 1.3. Spines were categorized as thin type if the spines exhibited a length-to-neck diameter ratio of more than 2.0 and a head-to-neck diameter ratio of <1.3 (Du et al., 2014). Spine density and morphology were measured separately for portions of three to five selected secondary dendrites per neuron.

### Neuronal synaptic density analysis

Cultured hippocampal neurons were fixed in 4% paraformaldehyde for 20 min after the neurons were permeabilized with 0.5% Triton X-100 in 0.1% sodium citrate and blocked with 10% goat serum. Up to 0.25 mL each of anti-MAP2 antibody (Abcam, 1:100) and anti-synaptophysin (Abcam, 1:200) was added to each chamber, followed by incubation with goat anti-rabbit and mouse secondary antibodies (Alexa Fluor 488, Alexa Fluor 594, Abcam, UK) for 30 min at 37°C. Images were taken under a laser-scanning confocal microscope (TCS-SPE, Leica, Germany) by using a 40× objective and excitation of 488 and 543 nm, and Z-stacks were gathered at increments of 0.67 μm. The *z*-stack images were compressed to a single image via the max projection method and analyzed using NIH ImageJ program.

### Statistical analysis

The results were processed for statistical analysis using SPSS (version 20.0 for Windows). The results are presented as the mean ± standard error of the mean (SEM). Statistical analyses were performed with one-way analysis of variance, followed by Fisher's protected least significant difference test for *post-hoc* comparisons. A value of *P* < 0.05 was considered significant.

## Results

### Geniposide ameliorated Aβ_1–42_-induced axonal mitochondrial abnormalities

Mitochondria are vital to the function of synapses because they supply energy for maintenance, calcium buffering, synaptic transmission, and vesicle release. Mitochondria are thought to be synthesized perinuclearly. Thus, they must be transferred from the soma to distal synapses through axons and dendrites via mitochondrial transport and constantly reconfigured to meet synaptic needs. Mitochondrial morphology is also dynamic and can be regulated through fusion and fission. An elongated morphology may confer bioenergetic advantages for ATP generation and dispersal (Skulachev, 2001). Therefore, the effects of geniposide on Aβ-induced abnormal axonal mitochondrial trafficking, distribution, and morphology were analyzed. A primary cultured hippocampal neuronal model was examined as previously reported because of the technical limitations on observing mitochondrial trafficking *in vivo* (Du et al., 2010). Axonal processes were selected for the quantitative analysis of mitochondrial length, density, distribution, and mobility because of their known morphologic and dynamic characteristics (Banker and Cowan, 1979; Du et al., 2010) and because of the significantly synaptic pathology of AD.

#### Geniposide attenuated Aβ_1–42_-induced axonal mitochondrial fragmentation

**Figure 1:**
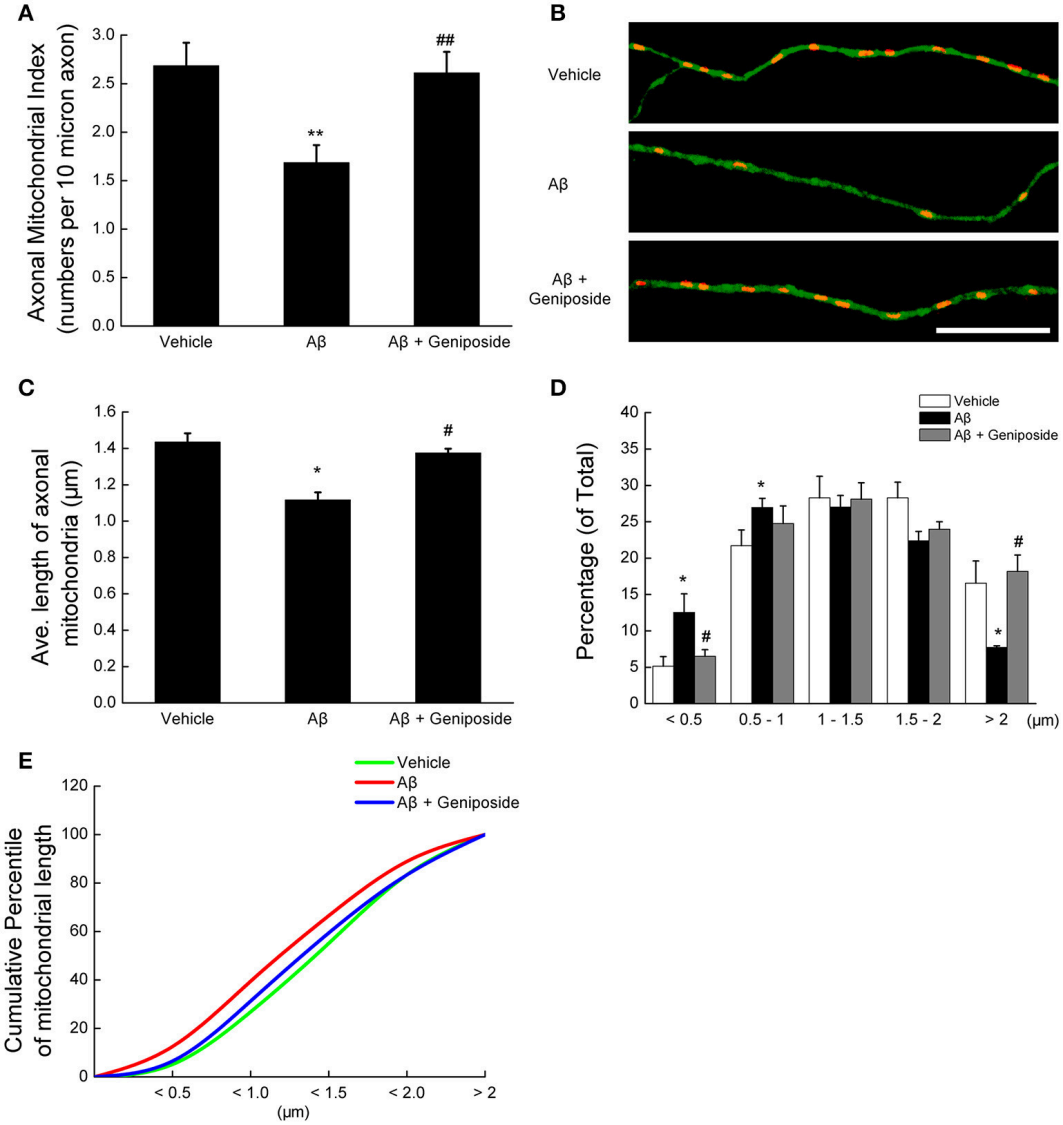
**Geniposide ameliorated the reduction in axonal mitochondrial density and length induced by oligomeric Aβ_1–42_ treatment. (A)** Axonal mitochondria from hippocampal neurons (14 DIV) were analyzed after 24 h of treatment with vehicle, oligomeric Aβ_1–42_ (200 nM), or oligomeric Aβ_1–42_+geniposide (10 μM). The axonal mitochondrial index (numbers per 10 μm of axon) was computed from three independent experiments. ^**^p < 0.01 vs. vehicle-treated group, ^##^*p* < 0.01 vs. Aβ-treated group. **(B)** Representative images of axonal mitochondrial distribution for the groups treated with vehicle, oligomeric Aβ_1–42_, or oligomeric Aβ_1–42_+geniposide. Double fluorescent staining with MitoTracker Red (red, mitochondrial marker) and Tau (green, axonal marker) was performed. Scale bar = 10 μm. **(C)** Average lengths of axonal mitochondria in the three groups. ^*^p < 0.05 vs. vehicle-treated group, ^#^*p* < 0.05 vs. Aβ-treated group. **(D)** Distribution of axonal mitochondrial length in the three groups. **(E)** Cumulative percentile of mitochondrial length in C. *n* = 3 independent cultures, eight axons per group.

First, axonal mitochondrial density was evaluated by counting the mitochondria (particles positive for MitoTracker Red) in each axonal process of the same length. As shown in Figures 1A,B, the axonal mitochondrial density was significantly lower (~37%) in neurons treated with Aβ_1–42_ than in vehicle-treated neurons (2.685 ± 0.237 vs. 1.685 ± 0.181 per 10 μm; *p* < 0.01). However, the axonal mitochondrial density of neurons pretreated with 10 μM geniposide was significantly higher than that of neurons treated with Aβ_1–42_ alone (2.611 ± 0.217 vs. 1.685 ± 0.181 per 10 μm; *p* < 0.01).

Second, the changes in mitochondrial morphology were detected by measuring the axonal mitochondrial length. Treatment with 200 nM oligomeric Aβ_1–42_ significantly decreased the average length of axonal mitochondria compared with hippocampal neurons treated with vehicle or geniposide (1.117 ± 0.049 μm in Aβ_1–42_-treated neurons vs. 1.435 ± 0.041 μm in vehicle-treated neurons or 1.375 ± 0.023 μm in geniposide- and Aβ_1–42_-treated neurons; *p* < 0.05; Figure 1C). The percentage of axonal mitochondria <0.5 μm in length in neurons exposed to Aβ was significantly higher than in neurons treated with vehicle or geniposide (Figure 1D), whereas the percentage of mitochondria more than 2.0 μm long decreased. Cumulative data indicated a leftward shift in the mitochondrial length of Aβ_1–42_-treated neurons (Figure 1E).

#### Geniposide ameliorated the Aβ_1–42_-induced impairment of axonal mitochondrial trafficking

**Figure 2:**
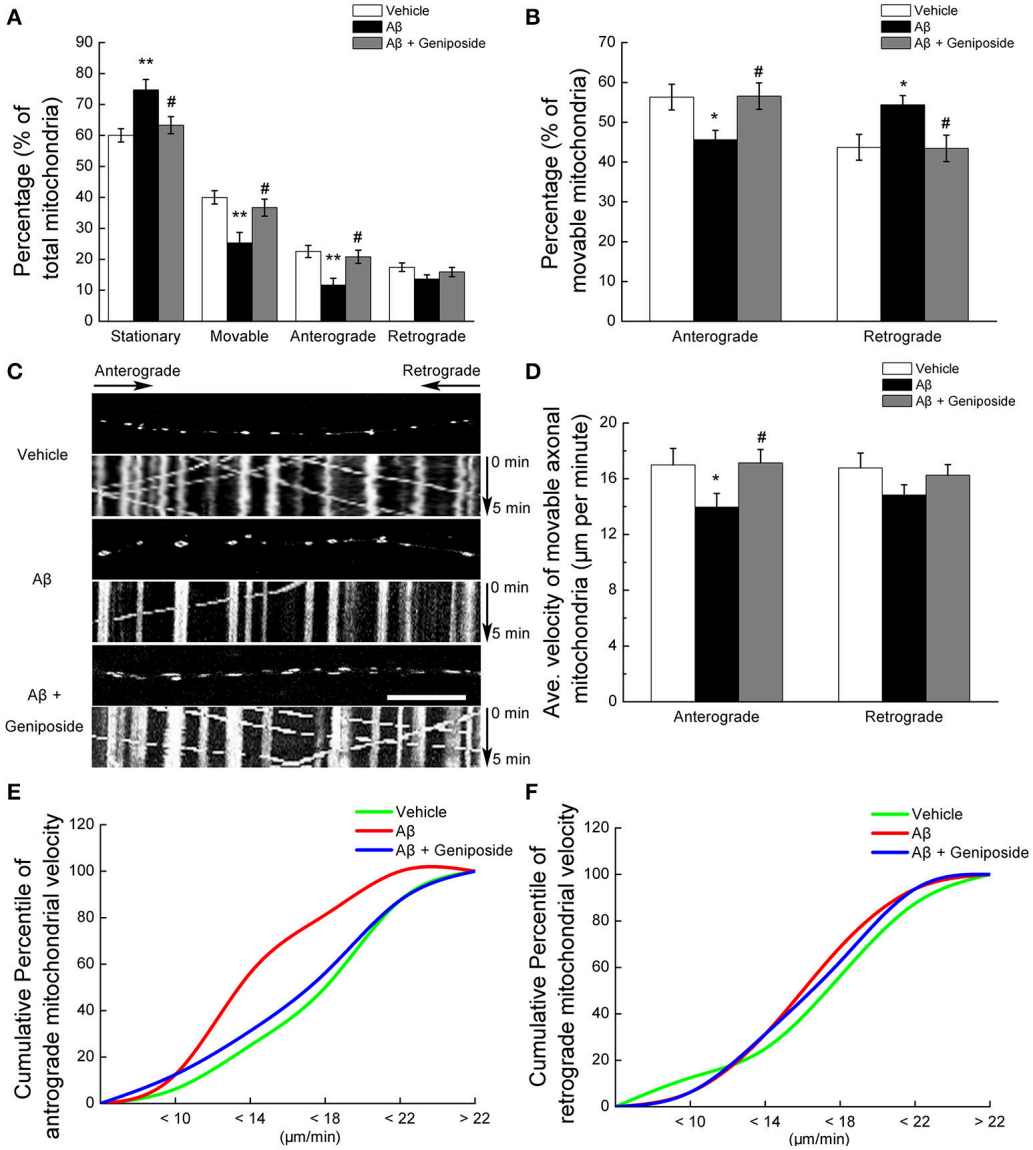
**Geniposide ameliorated the abnormal axonal trafficking of mitochondria induced by oligomeric Aβ_1–42_ treatment**. Axons from hippocampal neurons at 14 DIV were analyzed after 24 h of treatment with vehicle, oligomeric Aβ_1–42_ (200 nM), or oligomeric Aβ_1–42_+geniposide (10 μM). Fluorescent staining with MitoTracker Green (mitochondrial marker) was performed. **(A)** Percentages of stationary, movable, anterograde-moving, and retrograde-moving mitochondria were calculated compared with the numbers of total mitochondria. ^**^p < 0.01 vs. vehicle-treated group, ^#^*p* < 0.05 vs. Aβ-treated group. **(B)** Percentages of anterograde-moving and retrograde-moving mitochondria were calculated compared with the total numbers of movable mitochondria. ^*^p < 0.05 vs. vehicle-treated group, ^#^*p* < 0.05 vs. Aβ-treated group. **(C)** Calculations were based on analysis of kymographs. Representative kymographs of the axonal mitochondrial movement in the three experimental groups. Scale bar = 10 μm. **(D)** Average velocity of anterograde and retrograde transport of mitochondria for all movable mitochondria (μm/min) is shown. ^*^p < 0.05 vs. vehicle-treated group, ^#^*p* < 0.05 vs. Aβ-treated group. **(E,F)** Cumulative percentile of anterograde or retrograde mitochondrial velocity in D. *n* = 4 independent cultures, six axons per group.

Third, mitochondrial trafficking within hippocampal axonal processes was investigated. The percentage of stationary mitochondria among total mitochondria in Aβ_1–42_-treated neurons was significantly higher (by ~15%) that that in the neurons treated with vehicle (60.028 ± 2.154 vs. 74.695 ± 3.363%; *p* < 0.01, Figure 2A), and geniposide treatment produced a partial recovery of the percentage of stationary mitochondria (63.319 ± 2.761%; *p* < 0.05; Figure 2A). The percentage of mitochondria moving in either direction (anterograde and retrograde) in Aβ_1–42_-treated neurons was significantly lower than in vehicle-treated and geniposide-treated neurons (Figures 2A,C). The percentage of anterograde mitochondria among movable mitochondria in Aβ_1–42_-treated neurons slightly decreased compared with neurons treated with vehicle or geniposide (45.63 ± 2.324% in Aβ_1–42_-treated neurons vs. 56.3 ± 3.24% in vehicle-treated neurons or 56.55 ± 3.323% in geniposide- and Aβ_1–42_-treated neurons; *p* < 0.05; Figure 2B). By contrast, the percentage of retrograde mitochondria increased in Aβ_1–42_-treated neurons.

Fourth, the velocity of mitochondrial movement within axons was measured. Aβ_1–42_ treatment produced a greater detrimental effect on anterograde mitochondrial movement velocity than retrograde (Figure 2D). Aβ_1–42_ treatment decreased the anterograde velocity of axonal mitochondria by 18% (16.996 ± 1.18 μm/min in Aβ_1–42_-treated neurons vs. 13.965 ± 0.98 μm/min in vehicle-treated neurons; *p* < 0.05; Figures 2D,E), whereas the geniposide treatment decreased the toxicity of Aβ_1–42_ on the anterograde mitochondrial velocity (16.996 ± 1.18 μm/min in Aβ_1–42_-treated neurons vs. 17.134 ± 0.961 μm/min in geniposide- and Aβ_1–42_-treated neurons; *p* < 0.05). However, the retrograde transport velocity of axonal mitochondria did not differ significantly among the three groups (Figures 2D,F). Cumulative data displayed a leftward shift in the anterograde mitochondrial velocity (Figure 2E) but not in the retrograde mitochondrial velocity (Figure 2F) of Aβ_1–42_-treated neurons.

#### Geniposide attenuates Aβ_1–42_-induced neurites ROS elevation

**Figure 3:**
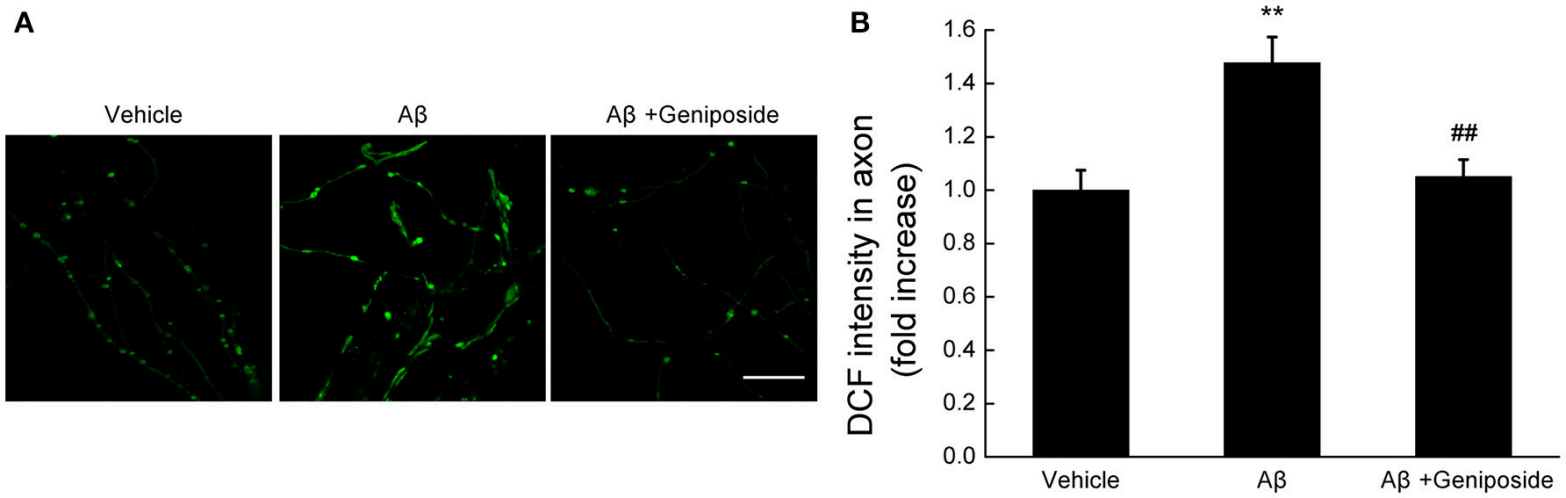
**Geniposide treatment attenuates the oligomeric Aβ-induced increase in neuritic ROS. (A)** Representative images of DCF staining of hippocampal neurites treated with vehicle, oligomeric Aβ_1–42_ (200 nM), or oligomeric Aβ_1–42_+geniposide (10 μM). Scale bar = 10 μm. **(B)** Quantification of DCF intensity in **(A)**. The intensity of DCF-labeled neurites was significantly increased in neurons treated with oligomeric Aβ_1–42_ for 24 h, while the geniposide treatment dramatically reduced the Aβ-induced ROS elevation. ^**^p < 0.01 vs. vehicle-treated group, ^##^*p* < 0.01 vs. Aβ-treated group. *n* = 4 independent cultures.

The mitochondrial ROS level was evaluated by measuring the fluorescence intensity of DCF within neurites. As shown in Figures 3A,B, the fluorescence intensity of DCF within the neurites of Aβ_1–42_-treated hippocampal neurons significantly increased (by ~48%), unlike the vehicle-treated or geniposide-treated neurons (1.0 ± 0.075 in Aβ_1–42_-treated neurons vs. 1.478 ± 0.097 in vehicle-treated neurons or 1.050 ± 0.065 in geniposide- and Aβ_1–42_-treated neurons; *p* < 0.01; Figure 3B). These results suggested that geniposide treatment significantly reduced the Aβ-induced increase in ROS in neurite mitochondria.

These data indicate that geniposide plays a protective role against the increased mitochondrial fragmentation, ROS elevation, and defective axonal mitochondrial trafficking induced by oligomeric Aβ_1–42_ in neurons.

### Geniposide alleviated Aβ_1–42_-induced synaptic damage in cultured neurons and an AD model

Axonal mitochondria are dynamic organelles, and their dynamics, trafficking, and docking are critical to maintain synaptic function and plasticity. The impairment of axonal mitochondrial may lead to synaptic loss and dysfunction. Various lines of evidence indicate that synaptic loss and deactivation are the biological bases of AD, and the accumulation of Aβ is an early event associated with synaptic and mitochondrial damage in AD. Thus, the density of synapses, the morphology of dendritic spines, and the levels of synapse-related proteins were analyzed to assess the contributions of abnormal mitochondrial fragmentation and trafficking to synaptic loss and dysfunction.

#### Geniposide protected against Aβ_1–42_-induced synaptic loss

**Figure 4:**
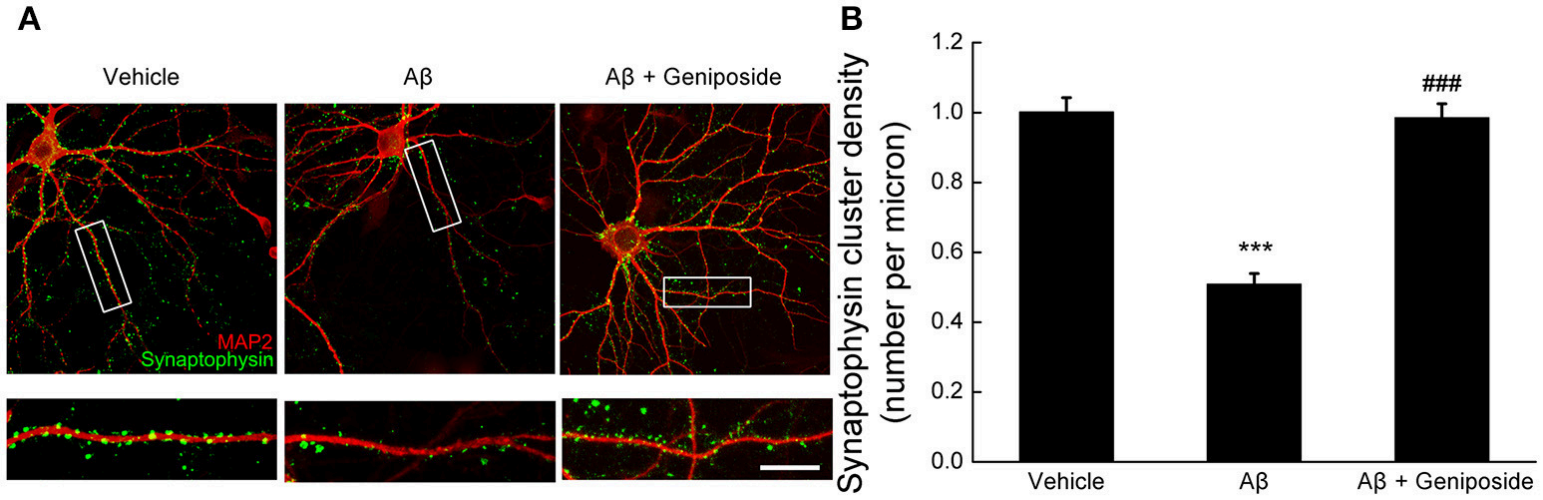
**Effect of geniposide on Aβ-induced synaptic loss**. Cultured hippocampal neurons at 14 DIV were analyzed after 24 h of treatment with vehicle, oligomeric Aβ_1–42_ (200 nM), or oligomeric Aβ_1–42_+geniposide (10 μM). Immunostaining with MAP2 (neuronal marker) and synaptophysin (synaptic marker) was performed. **(A)** Representative images for neurons and synapses treated with vehicle, oligomeric Aβ_1–42_, or oligomeric Aβ_1–42_+geniposide. Double immunostaining with MAP2 and synaptophysin. Scale bar = 10 μm. **(B)** Numbers of synaptophysin-positive clusters per micron of dendrites were significantly increased in Aβ_1–42_+geniposide-treated neurons compared with Aβ_1–42_-treated neurons. ^***^p < 0.001 vs. vehicle-treated group, ^###^*p* < 0.001 vs. Aβ-treated group. *n* = 4 independent cultures, eight neurons per group.

Synaptic density was measured by counting synaptophysin-positive clusters (green) on dendrites in order to determine the protective effect of geniposide on oligomeric Aβ_1–42_-induced synaptic loss. As shown in Figure 4A, there were significantly fewer synaptophysin-positive clusters in Aβ-treated neurons than in vehicle- or geniposide-treated neurons. The density of synapses in Aβ_1–42_-treated neurons was significantly lower (by ~49%) than in the vehicle-treated neurons (1.001 ± 0.041 per micron vs. 0.509 ± 0.030 per micron; *p* < 0.001, Figure 4B), whereas neurons pre-treated with geniposide (10 μM) showed a higher density of synapses than the Aβ_1–42_-treated group (0.985 ± 0.040 per micron vs. 0.509 ± 0.030 per micron; *p* < 0.001).

#### Geniposide rescued Aβ_1–42_-induced abnormal spine density and morphology

Dendritic spine density and morphology significantly changed in transgenic AD mouse models (Perez-Cruz et al., 2011; Penazzi et al., 2016); therefore, the spine phenotypes in cultured primary hippocampal neurons were investigated using CellTracker CM-DiI, and dendritic spines were categorized into three groups (mushroom, stubby, and thin).

**Figure 5:**
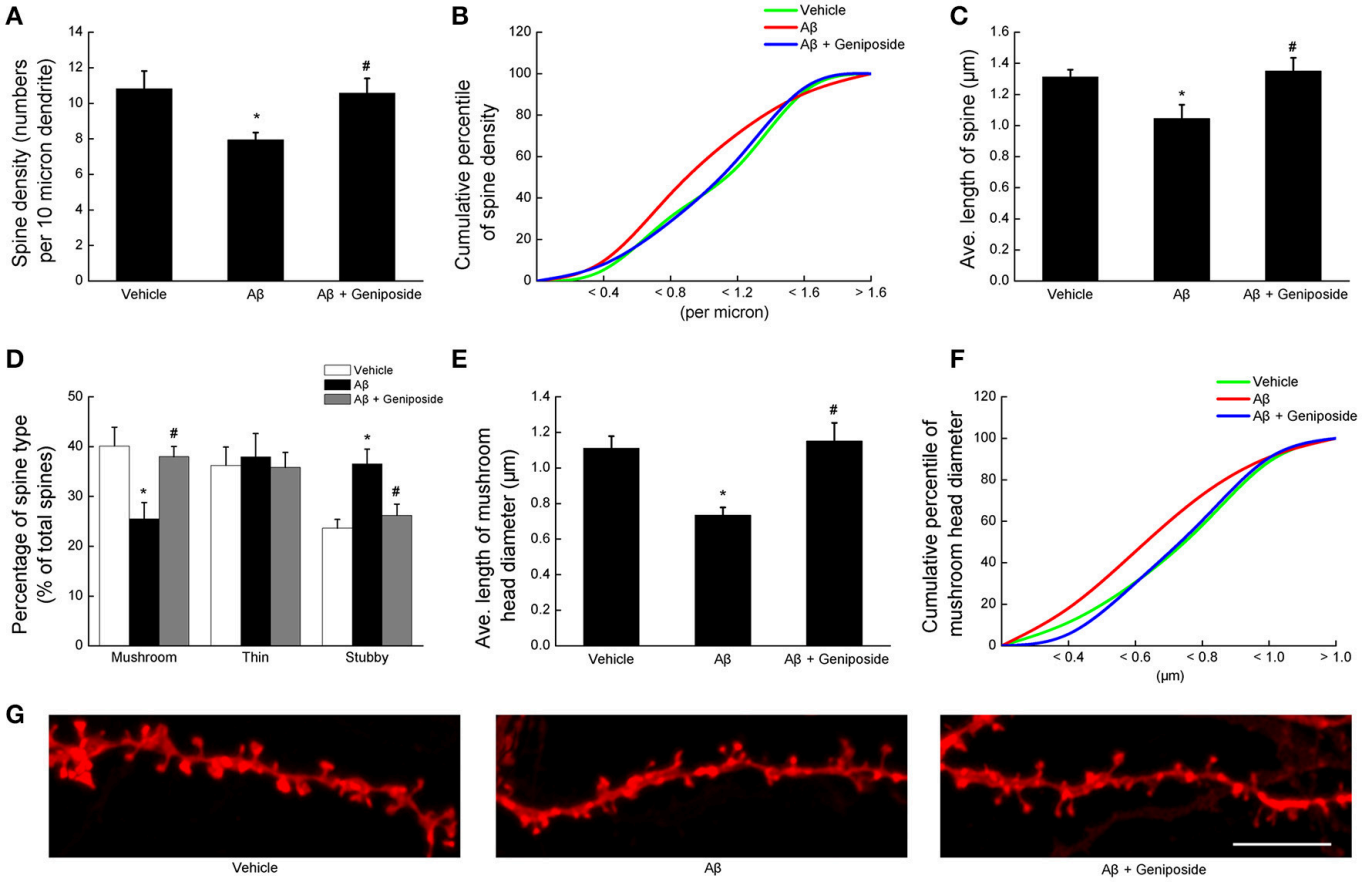
**Geniposide rescued the Aβ_1–42_-induced abnormal spine density and morphology**. Dendrites from hippocampal neurons at 14 DIV were analyzed after 24 h of treatment with vehicle, oligomeric Aβ_1–42_ (200 nM), or oligomeric Aβ_1–42_+geniposide (10 μM). Fluorescent staining with CellTracker CM-DiI was performed. **(A)** Quantification of total spine density per 10 μm of dendrite. **(B)** Cumulative percentile of spine density in **(A)**. **(C)** Average lengths of all spines in the three groups. **(D)** Percentages of the three spine types (mushroom, thin, and stubby) in total spines. **(E)** Average head diameter of mushroom spines. **(F)** Cumulative percentile of mushroom head diameter in **(E)**. **(G)** Representative images of DiI-labeled dendritic segments. Scale bar = 5 μm. ^*^P < 0.05 vs. vehicle-treated group, ^#^*p* < 0.05 vs. Aβ-treated group. *n* = 4 independent cultures, eight neurons per group.

First, the spine density was examined. As expected, Aβ_1–42_ treatment significantly decreased the spine density on dendrites, whereas geniposide-treated neurons showed notable recovery (7.95 ± 0.405 per 10 μm in Aβ_1–42_-treated neurons vs. 10.825 ± 0.994 per 10 μm in vehicle-treated neurons or 10.575 ± 0.826 per 10 μm in geniposide- and Aβ_1–42_-treated neurons; *p* < 0.05; Figure 5A). Cumulative data showed a substantial leftward shift in the spine density of Aβ_1–42_-treated neurons (Figure 5B).

Subsequently, the spine morphology was evaluated in terms of spine length and classification (mushroom, stubby, and thin). The morphology of the spines from Aβ_1–42_-treated neurons changed markedly from that of vehicle-treated neurons: the former appeared shorter and more stubby, with fewer mushroom-type spines than the latter (Figure 5G). Calculation of the average length of spines showed that Aβ_1–42_ treatment significantly decreased the spine length (by ~20%), whereas geniposide treatment reversed this pattern (Figure 5C). The quantitative analysis of dendritic spines in Aβ_1–42_-treated neurons showed that the percentage of mushroom-like spines significantly decreased (25.476 ± 3.308% in Aβ_1–42_-treated neurons vs. 40.129 ± 3.776% in vehicle-treated neurons or 37.977 ± 2.052% in geniposide- and Aβ_1–42_-treated neurons; *p* < 0.05; Figures 5D,G), but the percentage of stubby-like spines increased (36.548 ± 2.957% in Aβ_1–42_-treated neurons vs. 23.641 ± 1.755% in vehicle-treated neurons or 26.206 ± 2.268% in geniposide- and Aβ_1–42_-treated neurons; *p* < 0.05; Figures 5D,G). However, pretreatment with geniposide almost completely reversed the deleterious effect of Aβ_1–42_ on the percentages of stubby and mushroom spines. Geniposide considerably increased the average mushroom head diameter compared with Aβ_1–42_-treated neurons (1.152 ± 0.102 vs. 0.735 ± 0.043 μm; *p* < 0.05; Figure 5E). The plots of cumulative percentage curves clearly displayed a leftward shift in the mushroom head diameter of Aβ_1–42_-treated neurons (Figure 5F). These data indicated that geniposide treatment significantly preserved dendritic spine density and morphology, which may play a protective role in synaptic function.

#### Geniposide ameliorated the decrease in synapse-related proteins in cultured neurons and mice

**Figure 6:**
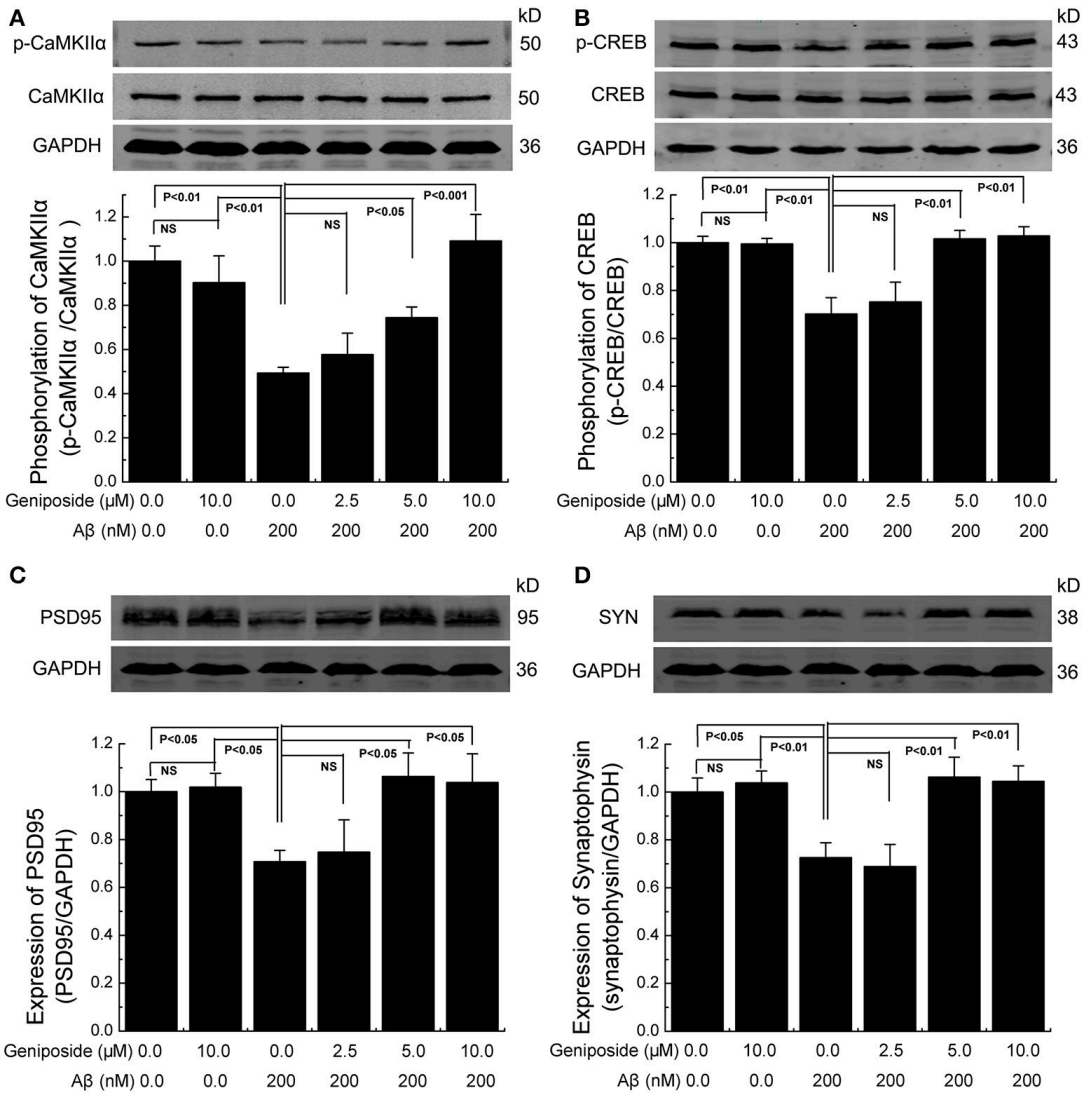
**Geniposide alleviated the decrease in synapse-related proteins in cultured primary hippocampal neurons. (A–D)** Western blot analysis of phospho-CaMKIIα and total-CaMKIIα **(A)**, phospho-CREB and total-CREB **(B)**, PSD-95 **(C)**, synaptophysin (SYN) **(D)**, and GAPDH in hippocampal neurons treated with the indicated concentrations of oligomeric Aβ_1–42_ or geniposide. Western blot of synaptophysin, PSD-95, and GAPDH were performed in the same membrane, therefore they share the same loading control (GAPDH). Quantification of p-CaMKIIα/CaMKIIα **(A)** p-CREB/CREB **(B)**, PSD-95/GAPDH **(C)**, and synaptophysin/GAPDH **(D)** was performed with the values from vehicle-treated neurons set as 1.0. Data are presented as the mean ± SEM. *n* = 4 independent cultures. NS, non-significant.

**Figure 7:**
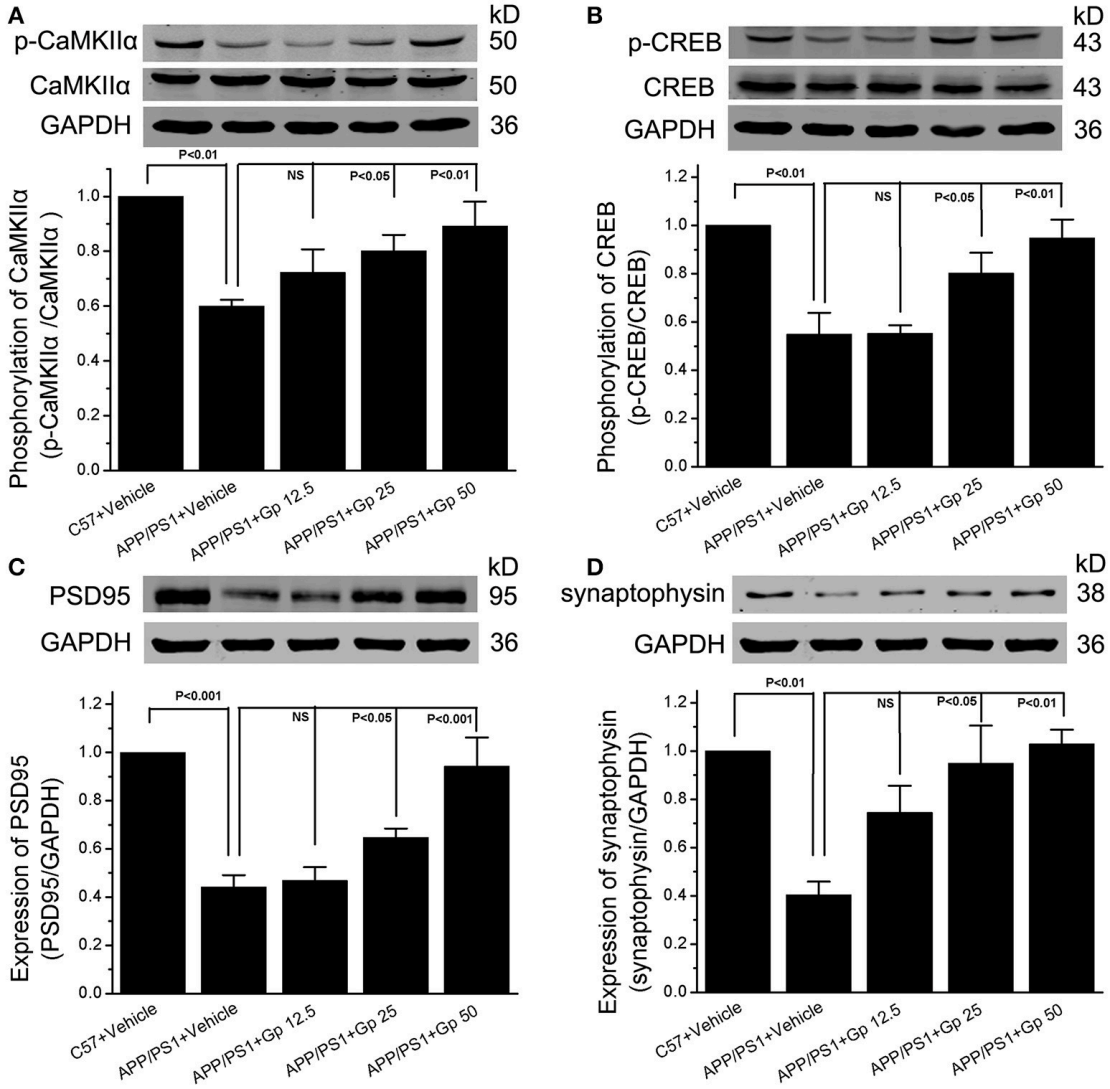
**Geniposide alleviated the decrease in synapse-related proteins in the hippocampi of APP/PS1 mice. (A–D)** Western blot analysis of phospho-CaMKIIα and total-CaMKIIα **(A)**, phospho-CREB, and total-CREB **(B)**, PSD-95 **(C)**, synaptophysin **(D)**, and GAPDH in the hippocampi of indicated group of mice with/without geniposide administration. Western blot of synaptophysin, PSD-95 and GAPDH were performed in the same membrane, therefore they share the same loading control (GAPDH). Quantification of p-CaMKIIα/CaMKIIα **(A)** p-CREB/CREB **(B)**, PSD-95/GAPDH **(C)**, and synaptophysin/GAPDH **(D)** was performed using the values from vehicle-treated WT mice as 1.0. Data are presented as the mean ± SEM. *n* = 6 mice for each group. Gp, mice treated with geniposide. NS, non-significant.

Synaptic plasticity is widely thought to be the basis of learning and memory formation. Synapse-related proteins are the foundation of synaptic plasticity. To investigate the effect of geniposide on the expression of synapse-related proteins, the levels of specific synaptic proteins in cultured primary hippocampal neurons were examined. The levels of synapse-related proteins significantly decreased in Aβ_1–42_-treated neurons relative to vehicle-treated neurons. As shown in Figure 6, the levels of p-CaMKIIα/CaMKIIα, p-CREB/CREB, synaptophysin, and PSD-95 in Aβ_1–42_-treated neurons were markedly lower than those in vehicle-treated neurons and significantly increased in neurons treated with various doses of geniposide (2.5, 5.0, and 10.0 μM geniposide; Figure 6). The expression of synapse-related proteins in the hippocampi of C57 and APP/PS1 (overexpressing Aβ) mice was examined further. Consistent with the data in cultured neurons, these results showed that the expression levels of p-CaMKII/CaMKIIα, p-CREB/CREB, synaptophysin, and PSD-95 were lower in vehicle-treated APP/PS1 mice than in vehicle-treated WT mice. Geniposide treatment (12.5, 25, and 50 mg/kg) of APP/PS1 mice also significantly increased the levels of the above proteins in a dose-dependent manner (Figure 7). These data indicate that the application of geniposide protects the expression of synapse-related proteins against the neurotoxic effects of Aβ in neurons and APP/PS1 mice.

## Discussion

There are evidence suggest that geniposide have multifaceted neuroprotective effects, such as ameliorating cholinergic deficit (Zhao et al., 2016b), increasing the expression of insulin-degrading enzyme (Zhang et al., 2015) and attenuating Aβ accumulation (Lv et al., 2015), inhibiting the signaling pathway of RAGE-MAPK and suppressing the production of proinflammatory mediators (Lv et al., 2014b, 2015), and protecting mitochondria by recovering ATP generation and mitochondrial membrane potential (Lv et al., 2014a; Zhao et al., 2016a). However, the protective effect of geniposide on Aβ induced mitochondrial transport and synaptic injury remains unclear.

The protective effect of geniposide against oligomeric Aβ_1–42_ in primary cultured hippocampal neurons was investigated in the current study. In consideration of the influence of overproduction of amyloid precusor protein (APP) and its metabolites in APP transgenic mice's neurons, we investigated the toxicity effect of Aβ by adding exogenous oligomeric Aβ_1–42_. Evidence suggests that RAGE mediates the transport of Aβ peptides across the cytomembrane from extracellular to intracellular (Deane et al., 2003; Takuma et al., 2009; Candela et al., 2010), including mitochondrial localization. Moreover, Aβ can be transported to the mitochondria via the TOM machinery and ER-mitochondrial crosstalk (Hansson Petersen et al., 2008; Hedskog et al., 2013).

In the present study, we demonstrate that geniposide protects cultured primary hippocampal neurons from the Aβ-induced impairment of axonal mitochondrial transport by improving axonal mitochondrial morphology, motility, and distribution, as well as rescuing anterograde mitochondrial movement, thus alleviating Aβ-induced synaptic injury by increasing the numbers of synapses and dendritic spines and the levels of synapse-related proteins, as well as attenuating the increase in ROS.

Axonal mitochondrial transport and synaptic mitochondrial distribution play crucial roles in synaptogenesis, synaptic transmission, and synaptic plasticity (Chang et al., 2006). Abnormal synaptic mitochondrial mobility and dynamics are responsible for synaptic failure. Considering the critical role of synaptic failure and neuronal dysfunction in AD pathogenesis, studies have investigated the mechanisms of synaptic mitochondrial perturbation that contribute to synaptic dysfunction (Du et al., 2008, 2010). These studies indicated that Aβ-induced abnormal axonal mitochondrial trafficking and synaptic mitochondrial dysfunction are responsible for the synaptic injury in AD.

Presynaptic terminals require mitochondria to handle calcium buffering, power the plasma membrane Ca^2+^-ATPase, and release Ca^2+^ to maintain post-tetanic potentiation (Medler and Gleason, 2002). Furthermore, synthesis of neurotransmitters, release of synaptic vesicles, outgrowth of axonal, and maintenance of synaptic plasticity also need mitochondria to supply high levels of ATP (Dillon and Goda, 2005). Therefore, decreased mitochondrial transport in axons likely impairs the delivery of organelles to synapses. Aβ has been reported to cause rapid and severe impairment of synaptic mitochondrial distribution and axonal mitochondrial mobility and thus to increase axonal mitochondrial fragmentation (Rui et al., 2006; Du et al., 2010; Calkins et al., 2011; Manczak et al., 2011). The current results regarding mitochondrial morphology and mobility indicated that geniposide ameliorated the effects of Aβ treatment that were induced by abnormal mitochondrial morphology and trafficking in axons by increasing the mitochondrial density and length and the proportions of movable and anterograde-transported mitochondria, as well as the average velocity of movable axonal mitochondria. These data suggest that geniposide may protect synaptic mitochondrial function from Aβ-induced impairment at an early stage.

Aβ-induced axonal and synaptic mitochondrial dysfunction is accompanied by synaptic degradation and is exhibited as the decrease of synapse-related proteins, the loss of dendritic spines and synapses, and morphological changes to spines. Synapse-related proteins constitute the structural and functional foundation of synapses. However, the expression levels of synapse-related proteins, such as synaptophysin, PSD-95, p-CaMKII, and p-CREB, are significantly decreased in the cortex and hippocampus of AD patients and of an AD mouse model (Gylys et al., 2004; Almeida et al., 2005; Zeng et al., 2015). Moreover, synaptic loss is the most severe condition of synaptic injury, and it manifests as the loss of dendritic spines and synapses in the brains of a transgenic AD mouse model and of AD patients. The density of dendritic spines significantly decreased in the cortex and hippocampus of several AD models (Yang et al., 2011; Du et al., 2014; Price et al., 2014), as well as in cultured primary hippocampal or cortical neurons treated with Aβ *in vitro* (Jo et al., 2011; Chen et al., 2015). In conclusion, the axonal and synaptic mitochondria are vulnerable and susceptible to various types of damage, leading to the synaptic degradation in AD. In the present study, geniposide-treatment significantly alleviated Aβ-induced synaptic injury by protecting the expression levels of synapse-related proteins, the density of dendritic spines and synapses, and the morphology of spines. Similar results were obtained for the expression of synapse-related proteins in the hippocampi of APP/PS1 mice.

In summary, the results demonstrated that geniposide protected primary neurons from Aβ-induced oxidative stress and from the acute impairment of axonal mitochondrial trafficking and morphology. This protective effect is associated with structural and functional changes in synapses. These findings indicate that axonal and synaptic mitochondria are vulnerable and susceptible to injury by Aβ at a low concentration, whereas geniposide can alleviate such injury. Thus, geniposide is a potential therapeutic agent that can be used to halt and prevent AD progression at an early stage.

## Author contributions

HZ, CZ, YW, and WZ designed the research; CZ, HZ, SD, CL, and ZL performed the research and analyzed data; and CZ, HZ, and WZ wrote the paper. All authors read and approved the final manuscript.

### Conflict of interest statement

The authors declare that the research was conducted in the absence of any commercial or financial relationships that could be construed as a potential conflict of interest.
